# Towards computable taxonomic knowledge: Leveraging nanopublications for sharing new synonyms in the Madagascan genus *Helictopleurus* (Coleoptera, Scarabaeinae)

**DOI:** 10.3897/BDJ.12.e120304

**Published:** 2024-06-14

**Authors:** Michele Rossini, Giulio Montanaro, Olivier Montreuil, Sergei Tarasov

**Affiliations:** 1 Finnish Museum of Natural History (LUOMUS), University of Helsinki, Helsinki, Finland Finnish Museum of Natural History (LUOMUS), University of Helsinki Helsinki Finland; 2 Department of Agronomy, Food, Natural resources, Animals and Environment (DAFNAE), University of Padova, Padova, Italy Department of Agronomy, Food, Natural resources, Animals and Environment (DAFNAE), University of Padova Padova Italy; 3 Muséum National d'Histoire Naturelle, Paris, France Muséum National d'Histoire Naturelle Paris France

**Keywords:** dung beetles, taxonomy, nomenclature, machine-readable data, SPARQL, ontology, Madagascar, extinction

## Abstract

**Background:**

Numerous taxonomic studies have focused on the dung beetle genus *Helictopleurus* d’Orbigny, 1915, endemic to Madagascar. However, this genus stilll needs a thorough revision. Semantic technologies, such as nanopublications, hold the potential to enhance taxonomy by transforming how data are published and analysed. This paper evaluates the effectiveness of nanopublications in establishing synonyms within the genus *Helictopleurus*.

**New information:**

In this study, we identify four new synonyms within *Helictopleurus*: *H.rudicollis* (Fairmaire, 1898) = *H.hypocrita* Balthasar, 1941 **syn. nov.**; *H.vadoni* Lebis, 1960 = *H.perpunctatus* Balthasar, 1963 **syn. nov.**; *H.halffteri* Balthasar, 1964 = *H.dorbignyi* Montreuil, 2005 **syn. nov.**; *H.clouei* (Harold, 1869) = *H.gibbicollis* (Fairmaire, 1895) **syn. nov.** *Helictopleurus* may have a significantly larger number of synonyms than currently known, indicating potentially inaccurate estimates about its recent extinction.

We also publish the newly-established synonyms as nanopublications, which are machine-readable data snippets accessible online. Additionally, we explore the utility of nanopublications in taxonomy and demonstrate their practical use with an example query for data extraction.

## Introduction

The dung beetle genus *Helictopleurus* d’Orbigny, 1915, which comprises 71 species (Montreuil 2005a), is endemic to Madagascar. It is mostly found in wet forests in the eastern part of the island, with a few species occurring in the drier western regions. Many *Helictopleurus* species are known solely from unique type specimens collected in poorly-sampled localities (e.g. *H.ambiguus* Paulian & Cambefort, 1991, *H.villiersi* Paulian & Cambefort, 1984 and *H.minutus* Paulian & Cambefort, 1984). In contrast, certain species have been described from localities that were intensively sampled at the beginning of the 20^th^ century, but have not been rediscovered since their description.

A recent revision of the *Helictopleurusfungicola* species-group (Rossini et al. 2021) has uncovered an instance of synonymy: *H.fungicola* (Fairmaire, 1899) = *H.pluristriatus* d'Orbigny, 1915. This discovery hinted at the possibility of further unrecognised synonyms within the genus, prompting us to conduct an additional examination of the type material.

The potential underestimation of synonyms in *Helictopleurus* suggests that future research could further reduce the number of recognised species in the genus. Consequently, the reported extinction of 50% of *Helictopleurus* species during the 20^th^ century (Hanski et al. 2007), which was attributed to deforestation, may not actually represent a genuine extinction phenomenon. Instead, it could be indicative of understudied taxonomy in the genus, where many species names might be synonyms. We address this issue in the discussion below.

We use nanopublications to share our results regarding the synonymies. A nanopublication represents a minimal unit of scientifically meaningful information that can be used to describe virtually anything (Kuhn and Dumontier 2017, Kuhn et al. 2021). It is written in a RDF (Resource Description Framework) format that computers can interpret. Through a decentralised network of services, nanopublications make the established synonyms instantly available online, adhering to the FAIR principles (Findable, Accessible, Interoperable, Reusable). This method of sharing information ensures that research findings are accessible to anyone, even to those who have not read the paper. In the subsequent sections, we discuss the utility of nanopublications for taxonomic nomenclature and provide an example query.

## Materials and methods

The studied specimens are deposited in the following institutes:

- NMPC: Národní National Museum, Prague, Czech Republic;
- MNHN: Muséum national d’Histoire naturelle, Paris, France;
- MZH: Finnish Museum of Natural History (LUOMUS), Helsinki, Finland;

Label data of the examined type specimens is provided in Darwin Core (DwC) format. We investigated only the genitalia of the type specimens whose identity could not be determined using external morphology alone. Specimens were photographed using a Canon EOS 5D Mark III and a Canon MP-E 65mm 2,8 1-5x macro lens. Images were edited in Adobe Photoshop. We used TaxonWorks to manage and retrieve the taxonomic history of the taxa treated in this study. The species distribution maps were generated in QGIS by assembling occurrence data from FinBIF (Finnish Biodiversity Information Facility), type and non-type specimens from the MNHN and NMPC. FinBIF data were retrieved in R, using the FinBIF package (Morris 2024). A Digital Elevation Model (DEM) for Madagascar was automatically generated with the OpenTopography DEM Downloader plugin as implemented in QGIS. Many of the collecting localities were georeferenced according to Viette (1991). Species occurrence data are provided in DwC format as a supplementary file (Suppl. material 1). Before including the data into the maps, the identity of the specimens was confirmed by morphological study and comparison with the types.

### Creating and querying nanopublications

The nanopublications were generated using the nanodash service, offered by Knowledge Pixels. Due to a collaborative effort between Pensoft Publishers and Knowledge Pixels, this service is accessible through the Biodiversity Data Journal (BDJ) portal. It enables the creation of nanopublications that can be seamlessly integrated with conventional publications for release in BDJ, a process we utilised in this research.

**Figure 1.**
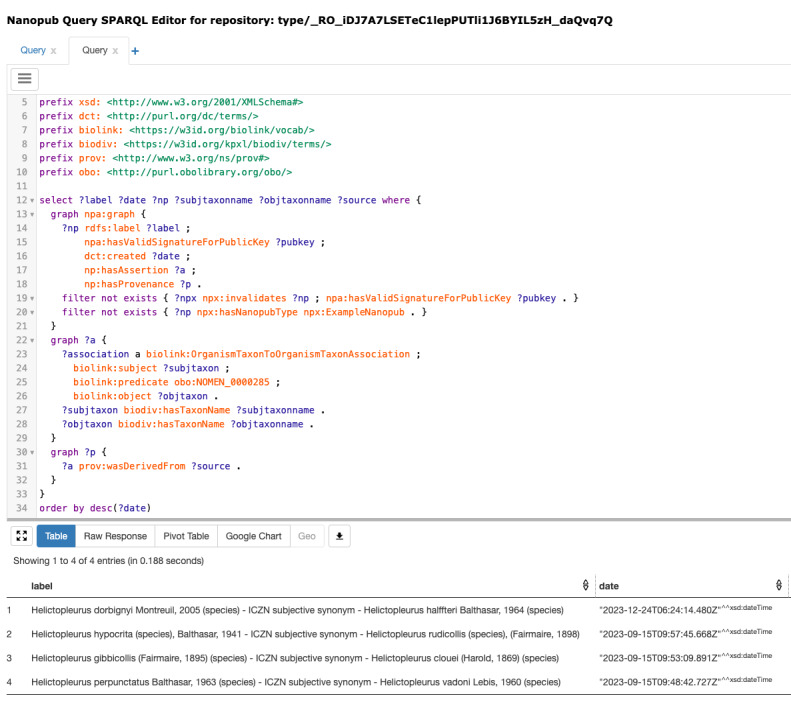
SPARQL query for extracting synonyms with the results of the query on the bottom.

Nanopublications can be queried using the endpoints at https://query.np.trustyuri.net. The exemplary SPARQL query, which is designed to retrieve all synonyms along with their corresponding valid names, is available via this link and the Supplementary Material (Suppl. material 2) (Fig. 1).

This query uses a SPARQL endpoint provided by Knowledge Pixels to select synonyms created using the nanodash service. Specifically, it searches for nanopublications of the type OrganismTaxonToOrganismTaxonAssociation.

## Taxon treatments

### Helictopleurus halffteri

Balthasar 1964

43A4641C-8844-5318-824D-032B591C0166

Helictopleurus halffteri Balthasar 1964: 623 (Figs 2b, 3d); Montreuil 2005b: 346;
*Helictopleurusdorbignyi* Montreuil 2005a: 123 (**syn. nov.**) (Figs 2a, 3d);

#### Materials

- **Type status:** Holotype. **Occurrence:** individualCount: 1; sex: female; lifeStage: adult; preparations: dry; disposition: in collection; occurrenceID: CFD3997A-2906-5551-BCF5-C12E9C1DFE2F; **Taxon:** scientificName: Helictopleurushalffteri Balthasar, 1963; namePublishedIn: Balthasar V. 1964. Neue Oniticellinen-Arten. (119. Beitrag zur Kenntnis der Scarabaeoidea (Coleoptera)). Beiträge zur Entomologie, 14(5-6):619–624; class: Insecta; order: Coleoptera; family: Scarabaeidae; genus: Helictopleurus; specificEpithet: halffteri; taxonRank: species; scientificNameAuthorship: Balthasar, 1963; nomenclaturalCode: ICZN; taxonomicStatus: accepted; **Location:** continent: Africa; country: Madagascar; locality: Antsianaka; **Identification:** identifiedBy: V. Balthasar; dateIdentified: 1963; **Event:** eventDate: 1893-07/12; year: 1893; verbatimEventDate: 2^e^ Semestre 1893; **Record Level:** institutionCode: NMPC; basisOfRecord: PreservedSpecimen
- **Type status:** Holotype. **Occurrence:** individualCount: 1; sex: male; lifeStage: adult; preparations: dry; disposition: in collection; otherCatalogNumbers: MALP0657; occurrenceID: 0026A7F3-DD0E-5D00-990A-33F6FE3C919F; **Taxon:** scientificName: Helictopleurusdorbignyi Montreuil, 2003; namePublishedIn: Montreuil O. 2005. Nouveaux *Helictopleurus* d’Orbigny, 1915 de Madagascar et révision du «groupe semivirens» sensu Lebis, 1960. Zoosystema, 27(1):123-135; class: Insecta; order: Coleoptera; family: Scarabaeidae; genus: Helictopleurus; specificEpithet: dorbignyi; taxonRank: species; scientificNameAuthorship: Montreuil, 2005; nomenclaturalCode: ICZN; taxonomicStatus: subjective synonym; **Location:** continent: Africa; country: Madagascar; locality: Antsianaka, lac Alaotra; verbatimLocality: Antsianaka et lac Alaotra; **Identification:** identifiedBy: O. Montreuil; dateIdentified: 2003; **Event:** eventDate: 1889-07/12; year: 1889; verbatimEventDate: 2^e^ Semestre 1889; **Record Level:** institutionCode: MNHN; basisOfRecord: PreservedSpecimen
- **Type status:** Lectotype. **Occurrence:** individualCount: 1; sex: male; lifeStage: adult; preparations: dry; disposition: in collection; otherCatalogNumbers: MALP0652; occurrenceID: F41F1258-D172-5CFC-AF96-A9612C20D073; **Taxon:** scientificName: Helictopleurus seminiger d'Orbigny, 1915; namePublishedIn: d'Orbigny, H. (1915) Synopsis d’un genre nouveau d’Oniticellides (Scarabaeidae Coprini) spécial à Madagascar. *Annales de la Société entomologique de France*, 84, 402–434.; class: Insecta; order: Coleoptera; family: Scarabaeidae; genus: Helictopleurus; specificEpithet: seminiger; taxonRank: species; scientificNameAuthorship: d'Orbigny, 1915; nomenclaturalCode: ICZN; taxonomicStatus: accepted; **Location:** continent: Africa; country: Madagascar; locality: Antsianaka; verbatimLocality: Antsianaka; **Identification:** identifiedBy: O. Montreuil; dateIdentified: 2003; **Event:** eventDate: 1892-01/06; year: 1892; verbatimEventDate: 1re Semestre 1892; **Record Level:** institutionCode: MNHN; basisOfRecord: PreservedSpecimen

#### Diagnosis

Within the *semivirens* group, *H.halffteri* may be related to *H.seminiger*. Males can be distinguished by the clypeal tubercle simple (laterally connected to two ridges in *H.seminiger*) and the frontoclypeal carina trituberculate (simple in *H.seminiger*). No female specimens of *H.seminiger* are known, as the type series includes the male lectotype only (see "Taxon discussion" below).

#### Distribution

Antsianaka and Lac Alaotra (Fig. 3d).

#### Taxon discussion

The *Helictopleurussemivirens* species-group includes 10 species: *H.aeneoniger* Lebis, 1960, *H.cambeforti* Montreuil, 2005, *H.halffteri* Balthasar, 1964, *H.heidiae* Montreuil, 2007, *H.niger* d’Orbigny, 1915, *H.pseudoniger* Montreuil, 2005, *H.seminiger* d’Orbigny, 1915, *H.semivirens* d’Orbigny, 1915, *H.steineri* Paulian & Cambefort, 1991 and *H.tamatavensis* Montreuil, 2005 (Montreuil 2005a).

*Helictopleurushalffteri* shares exclusive phenotypic traits with the members of the group, such as head ogival-shaped and elongated (unique within *Helictopleurus*); head of male often with clypeal tubercles, frontoclypeal carina and frontal tubercles and/or curved carina; dorsal side of the body very polished, fairly dark, dull to feebly shining; elytra visibly longer than pronotum with respect to the majority of *Helictopleurus* species.

Balthasar (1964) described *H.halffteri* on a single female specimen collected by the Perrot brothers in 1893 in Antsianaka and, after its description, the species name appeared only in one checklist (Montreuil 2005a). On studying the *Helictopleurus* of the *semivirens* group, Montreuil (2005a) discovered that the type series of *H.seminiger*, which allegedly consisted of one male and one female specimen, included instead two distinct species. Actually, the specimen that d’Orbigny (1915) identified as female turned out to be a male and was designated by Montreuil (2005a) as the holotype of the new *H.dorbignyi*. Therefore, the type series of *H.seminiger* currently consists of a unique male designated as the lectotype (Montreuil 2005a).

The type series of *H.dorbignyi* is composed of the male holotype collected in Antsianaka, lake Alaotra and multiple male and female paratypes collected further south, in the Ranomafana National Park and nearby localities (Fianarantsoa) (Fig. 3d). On studying the external morphology and genitalia of the type specimens of *H.dorbignyi* deposited in the MNHN, we realised that the type series is composed of at least two distinct species: one represented by the male holotype alone; the other represented by several males and females collected in Ranomafana. Therefore, in the light of the abovementioned facts and after examination of the holotype of *H.halffteri*, we conclude that:

(i) the morphology of the female holotype of *H.halffteri* and its type locality suggest that this specimen is conspecific with the male holotype of *H.dorbignyi*. Thus, *H.dorbignyi* is established as junior subjective synonym of *H.halffteri* (*H.dorbignyi* **syn. nov.**).

(ii) The study of the female genitalia of *H.halffteri* and female paratypes of *H.dorbignyi* collected in Ranomafana indicates that they belong to different species. Therefore, a new *Helictopleurus* species from Ranomafana is waiting formal description.

Interestingly, the unique type specimen of *H.seminiger* was collected from Antsianaka – which is the same type locality as *H.halffteri* – and the species has never been recollected since 1892. These circumstances would make the identity of this taxon questionable. However, during our visit to the Paris museum, we could not study the genitalia of the lectotype of *H.seminiger*. Possibly, the differences observed on the cephalic processes between *H.seminiger* and *H.halffteri* represent a simple variation of a same species. An extensive review of the *semivirens* group would help to sort out unresolved questions that still hamper the correct identification of these cryptic taxa.

### Helictopleurus rudicollis

(Fairmaire, 1898)

4D5F12BE-1371-5701-8FA2-B96CA7D9BB02

*Oniticellusrudicollis* Fairmaire 1898: 399
*Liatongusrudicollis*: *Fairmaire 1901*: 134 (new combination);
*Helictopleurusrudicollis*: d’Orbigny 1915: 418 (new combination); Gillet and Boucomont 1927: 111; Balthasar 1941: 89; Lebis 1960: 72; Paulian 1986: 106; Montreuil 2005a: 133;
*Helictopleurus hypocrita Balthasar 1941*: 134 (**syn. nov.**) (Fig. 2c) ; Montreuil 2005a: 133; Bezdĕk and Hájek 2012: 321;

#### Materials

- **Type status:** Syntype. **Occurrence:** individualCount: 1; sex: male; lifeStage: adult; preparations: dry; disposition: in collection; occurrenceID: 48AD10D1-BDF2-5606-9926-550DC3BF2A4D; **Taxon:** scientificName: Oniticellusrudicollis Fairmaire, 1898; acceptedNameUsage: Helictopleurusrudicollis; parentNameUsage: Scarabaeinae; namePublishedIn: Fairmaire L. (1898) Matériaux pour la faune coléoptérique de la région malgache (6e note). *Annales de la Société entomologique de Belgique*, 42, 390–439; class: Insecta; order: Coleoptera; family: Scarabaeidae; genus: Helictopleurus; specificEpithet: rudicollis; taxonRank: species; scientificNameAuthorship: Fairmaire, 1898; nomenclaturalCode: ICZN; taxonomicStatus: accepted; **Location:** continent: Africa; country: Madagascar; locality: Antsianaka; **Identification:** identifiedBy: L. Fairmaire; **Event:** eventDate: 1892-01/06; year: 1892; verbatimEventDate: 1er Semestre 1892; **Record Level:** institutionCode: MNHN; basisOfRecord: PreservedSpecimen
- **Type status:** Holotype. **Occurrence:** individualCount: 1; sex: male; lifeStage: adult; preparations: dry; disposition: in collection; occurrenceID: C866E3F5-DE1C-5A4A-8ACA-AF935D19F874; **Taxon:** scientificName: Helictopleurus hypocrita Balthasar, 1941; namePublishedIn: Balthasar V. 1941. Eine reihe von neuen coprophagen scarabaeiden (67. beitrag zur kenntnis der235 scarabaeidae, col. Mitteilungen der Munchen Entomologische Gesellschaft 31 (1): 164‑184.; class: Insecta; order: Coleoptera; family: Scarabaeidae; genus: Helictopleurus; specificEpithet: hypocrita; taxonRank: species; scientificNameAuthorship: Balthasar, 1941; nomenclaturalCode: ICZN; taxonomicStatus: subjective synonym; **Location:** continent: Africa; country: Madagascar; **Identification:** identifiedBy: V. Balthasar; **Record Level:** institutionCode: NMPC; basisOfRecord: PreservedSpecimen
- **Type status:** Holotype. **Occurrence:** individualCount: 1; sex: male; lifeStage: adult; preparations: dry; disposition: in collection; otherCatalogNumbers: MALP0550; occurrenceID: F8B71464-2E59-53F9-B084-F77380DA3C9B; **Taxon:** scientificName: Helictopleurusfurcicornis Lebis, 1960; namePublishedIn: Lebis E. 1960. Insectes, coléoptères Scarabaeidae, Helictopleurina. Faune de Madagascar, 11, 25–130; class: Insecta; order: Coleoptera; family: Scarabaeidae; genus: Helictopleurus; specificEpithet: furcicornis; taxonRank: species; scientificNameAuthorship: Lebis, 1960; nomenclaturalCode: ICZN; taxonomicStatus: valid; **Location:** continent: Africa; country: Madagascar; **Record Level:** institutionCode: MNHN; basisOfRecord: PreservedSpecimen
- **Type status:** Holotype. **Occurrence:** individualCount: 1; sex: male; lifeStage: adult; preparations: dry; disposition: in collection; otherCatalogNumbers: MALP531; occurrenceID: C36B8CD0-9D24-50D5-9BF2-6CD92F1F601F; **Taxon:** scientificName: Helictopleurus splendidus d'Orbigny, 1915; namePublishedIn: d'Orbigny H. 1915. Synopsis d’un genre nouveau d’Oniticellides (Scarabaeidae Coprini) spécial à Madagascar. Annales de la Société entomologique de France, 84, 402–434; class: Insecta; order: Coleoptera; family: Scarabaeidae; genus: Helictopleurus; specificEpithet: splendidus; taxonRank: species; scientificNameAuthorship: d'Orbigny, 1915; nomenclaturalCode: ICZN; taxonomicStatus: valid; **Location:** continent: Africa; country: Madagascar; verbatimLocality: Fianarantsoa; **Record Level:** institutionCode: MNHN; basisOfRecord: PreservedSpecimen
- **Type status:** Paratype. **Occurrence:** individualCount: 1; sex: male; lifeStage: adult; preparations: dry; disposition: in collection; otherCatalogNumbers: MALP532; occurrenceID: 13D0EB26-F1D1-5DCE-97EA-261312FC473C; **Taxon:** scientificName: Helictopleurus splendidus d'Orbigny, 1915; namePublishedIn: d'Orbigny H. 1915. Synopsis d’un genre nouveau d’Oniticellides (Scarabaeidae Coprini) spécial à Madagascar. Annales de la Société entomologique de France, 84, 402–434; class: Insecta; order: Coleoptera; family: Scarabaeidae; genus: Helictopleurus; specificEpithet: splendidus; taxonRank: species; scientificNameAuthorship: d'Orbigny, 1915; nomenclaturalCode: ICZN; taxonomicStatus: valid; **Location:** continent: Africa; country: Madagascar; verbatimLocality: Bémarivo, Région Est; **Record Level:** institutionCode: MNHN; basisOfRecord: PreservedSpecimen

#### Diagnosis

*Helictopleurusrudicollis* belongs to the *rudicollis* group and can be readily distinguished from other members of the group, *H.rubricollis* and *H.cribricollis*, by the pronotum and elytra metallic-green and shiny and elytra with lighter spots (elytra dark, dull, not metallic and without spots in both *H.rubricollis* and *H.cribricollis*). The separation of *H.rudicollis* from *H.splendidus* is still unclear, as the male of the latter was never described. Nonetheless, the following female characters could help in separating the two species: punctation of posterior third of pronotum evenly distributed in *H.rudicollis* (sparser in *H.splendidus*); lateral pronotal angles more expanded laterally in *H.splendidus*; median tubercle of frontal carina simple in *H.rudicollis* (bilobate in *H.splendidus*).

#### Distribution

This species is widely distributed across eastern Madagascar, from the Sambava to Ifanadiana District (Fig. 3a).

#### Taxon discussion

Balthasar (1941) described *H.hypocrita* on a unique specimen, but no exact collecting locality was given. Without checking the holotype, Lebis (1960) assigned this species to the *giganteus* group, suggesting that the original description could refer either to *H.undatus* (Olivier, 1789) or *H.rudicollis*. Ever since, *H.hypocrita* appeared only in one checklist (Montreuil 2005a). In this study, we investigated and compared the external and genital morphology of the holotype of *H.hypocrita* with the type specimens of the members of the *rudicollis* group and we eventually concluded that *H.hypocrita* is a junior subjective synonym of *H.rudicollis* (*H.hypocrita* **syn. nov.**).

The *Helictopleurusrudicollis* group includes four species (Lebis 1960, Montreuil 2005b): *H.rudicollis*, *H.cribricollis* Lebis, 1960, *H.rubricollis* Lebis, 1960 and *H.splendidus* d’Orbigny, 1915. However, after examination of the syntypes of *H.furcicornis* Lebis, 1960, we deem that this species too could be considered an additional member of the same group. *Helictopleurusfurcicornis* shares several characters with *H.rudicollis*: body metallic-green; elytra with a series of orange-brownish spots; long yellow setae along the lateral edges of the pronotum; tip of the parameres strongly curved downwards. However, the two species differ in the shape of the male frontal horn (conical in *H.rudicollis*; wider, with parallel sides and distally emarginated in *H.furcicornis*), pronotal punctuation (denser in *H.rudicollis*) and lighter spots on elytra (single transversal row in *H.rudicoliis*; elytral interval 4 with two additional spots, one in proximity of the base, one close to the apex).

*Helictopleurusrudicollis* was described from Tamatave and Antsianaka, which correspond to today’s Toamasina and Lac Alaotra, respectively, both located in central-eastern Madagascar (Viette 1991). Instead, the type series of *H.splendidus* includes specimens from Bémarivo (probably referring to a locality along the Bemarivo River, north-eastern Madagascar), Fianarantsoa and Fort Dauphin (central-eastern and south-eastern Madagascar, respectively) (d’Orbigny 1915) (Fig. 3a). Despite the multiple dung beetle surveys carried out over the last 20 years in the type and nearby localities, no additional specimens of *H.splendidus* have been captured or identified. On the other hand though, *H.rudicollis* is considered one of the most abundant species in these localities (Rahagalala et al. 2009). Therefore, given that the two species are morphologically very similar, the above statements raise a series of questions that need further investigation: (i) Have all the "*H.rudicollis*" specimens collected so far been identified correctly? (ii) is *H.splendidus* a valid species? (iii) If so, maybe this latter species has become extinct?

During a recent collecting expedition in Madagascar (February 2022), we collected a series of conspecific specimens in Mandraka Park (-18.914266, 47.92477098), Analamazoatra (-18.939524, 48.418760) and Mantadia (-18.84549399, 48.42834902) National Parks, including one male, that may belong to *H.splendidus*. However, a detailed comparison of the morphology of these specimens with the types of *H.splendidus* and *H.rudicollis* is needed to confirm this identification and the taxonomic status of *H.splendidus*.

### Helictopleurus vadoni

Lebis, 1960

92CAD555-5C00-5B31-8F3F-08B22AC28256

*Helictopleurusvadoni* Lebis 1960: 99; Paulian 1986: 107; Montreuil 2005a: 134;
*Helictopleurusparvulus* Frey 1975: 305; Paulian 1986: 107 (synonymy);
*Helictopleurusperpunctatus* Balthasar 1963: 292 (**syn. nov.**) (Fig. 2d); Montreuil 2005b: 376;

#### Materials

- **Type status:** Paratype. **Occurrence:** individualCount: 1; sex: female; lifeStage: adult; preparations: dry; disposition: in collection; occurrenceID: 4D23D8E1-C5F7-592F-A169-29C7092BFAD6; **Taxon:** scientificName: Helictopleurusperpunctatus Balthasar, 1963; namePublishedIn: Balthasar V. 1963. Neue arten der familie scarabaeidae. Acta Societatis Entomologicae Cechosloveniae, 60(4):284–295.; class: Insecta; order: Coleoptera; family: Scarabaeidae; genus: Helictopleurus; specificEpithet: perpunctatus; taxonRank: species; scientificNameAuthorship: Balthasar, 1963; nomenclaturalCode: ICZN; taxonomicStatus: subjective synonym; **Location:** continent: Africa; country: Madagascar; **Identification:** identifiedBy: V. Balthasar; dateIdentified: 1960; **Event:** eventDate: 1954-07; year: 1954; **Record Level:** institutionCode: NMPC; basisOfRecord: PreservedSpecimen
- **Type status:** Holotype. **Occurrence:** recordedBy: Vadon; individualCount: 1; sex: male; lifeStage: adult; preparations: dry; disposition: in collection; otherCatalogNumbers: MALP081; occurrenceID: 1ECAEFE1-3A1C-5AEB-84CD-1EC204E0CA16; **Taxon:** scientificName: Helictopleurusvadoni Lebis, 1960; namePublishedIn: Lebis, E. 1960. Insectes, coléoptères Scarabaeidae, Helictopleurina. *Faune de Madagascar*, 11:25–130; class: Insecta; order: Coleoptera; family: Scarabaeidae; genus: Helictopleurus; specificEpithet: vadoni; taxonRank: species; scientificNameAuthorship: Lebis, 1960; nomenclaturalCode: ICZN; taxonomicStatus: valid; **Location:** continent: Africa; country: Madagascar; verbatimLocality: Maroantsetra; **Identification:** identifiedBy: E. Lebis; **Event:** eventDate: 1944-9; year: 1944; **Record Level:** institutionCode: MNHN; basisOfRecord: PreservedSpecimen
- **Type status:** Paratype. **Occurrence:** recordedBy: Vadon; individualCount: 1; lifeStage: adult; preparations: dry; disposition: in collection; otherCatalogNumbers: MALP0820; occurrenceID: 67B9F2FB-62F6-5FCA-8F4D-166C7DD57E70; **Taxon:** scientificName: Helictopleurusvadoni Lebis, 1960; namePublishedIn: Lebis, E. 1960. Insectes, coléoptères Scarabaeidae, Helictopleurina. *Faune de Madagascar*, 11:25–130; class: Insecta; order: Coleoptera; family: Scarabaeidae; genus: Helictopleurus; specificEpithet: vadoni; taxonRank: species; scientificNameAuthorship: Lebis, 1960; nomenclaturalCode: ICZN; taxonomicStatus: valid; **Location:** continent: Africa; country: Madagascar; verbatimLocality: Ankovana; **Identification:** identifiedBy: E. Lebis; **Event:** eventDate: 1945-8; year: 1945; **Record Level:** institutionCode: MNHN; basisOfRecord: PreservedSpecimen

#### Diagnosis

*Helictopleurusvadoni* is readily distinguished from congeneric species by the following combination of characters: elytra dark, with metallic tinges and characteristic brownish-orange spots mostly concentrated on the basal half of the intervals 1–5; elytral intervals with erected setae; pronotum metallic-green, shiny, densely and evenly punctated and setose, discal punctures coarser.

#### Distribution

This species occurs in the Sambava and Maroantsetra Districts, north-eastern Madagascar (Fig. 3c).

#### Taxon discussion

*Helictopleurusperpunctatus* was described on two female specimens, without exact collecting locality. According to Balthasar (1963), the holotype is in J. Schulze’s collection (probably in the Museum für Naturkunde, Berlin), but we could not examine it. In this study, instead, we investigated the morphology of the paratype (NMPC), which clearly indicates that *H.perpunctatus* is a female of *H.vadoni*. Given the original description and Balthasar's expertise on scarabs, we believe that both the holotype and paratype are conspecific. Therefore, *H.perpunctatus* is established as the junior subjective synonym of *H.vadoni* (*H.perpunctatus* **syn. nov.**).

Balthasar (1963) states that his conclusions on *H.perpunctatus* was drawn after consultation of the keys from d’Orbigny (1915). Therefore, it is likely that he overlooked the *Helictopleurus* that meanwhile were described by Lebis (1960). In so doing, Balthasar considers *H.perpunctatus* a close relative of *H.infimus* (Fairmaire, 1901) and provides a list of characters to separate the two species.

Lebis (1960) included *H.vadoni* in the *fungicola-viridiflavus* group, along with seven additional species. These *Helictopleurus* were subsequently separated into two groups: *fungicola* and *viridiflavus* (Montreuil 2005a). Today, the *viridiflavus* group comprises the following species (Montreuil 2005b): *H.viridiflavus* (Fairmaire, 1898), *H.vadoni*, *H.infimus* (Fairmaire, 1901), *H.carbonarius* Lebis, 1960, *H.minutus* Paulian & Cambefort, 1984, *H.villiersi* Paulian & Cambefort, 1984 and *H.ambiguus* Paulian & Cambefort, 1991. The majority of these *Helictopleurus* seems to be strictly associated with dry and semi-arid regions of western Madagascar. The dung beetle fauna of these areas has been poorly investigated and many of these *Helictopleurus* are only known from the type series, which often consist of single specimens.

*Helictopleurusvadoni* and *H.viridiflavus* are the only members of the group that occur in the humid-most eastern Madagascar, with occasional incursions into drier regions (Fig. 3c). *Helictopleurusviridiflavus* is a very distinct species, with elytra brownish and without spots; pronotum metallic-green to coppery, with elongate and very dense punctures.

### Helictopleurus clouei

(Harold, 1869)

1838A91C-EEB2-54F7-B482-C2BE73791BCF

*Oniticellusclouei* Harold 1869: 68; Harold 1880: 155; Fairmaire 1895: 13; Fairmaire 1898: 471;
*Helictopleurusclouei*: d’Orbigny 1915: 428 (new combination) (Figs 2e, 3b); Lebis 1960: 111; Gillet and Boucomont 1927: 110; Paulian 1986: 104; Montreuil 2005a: 134;
*Oniticellusgibbicollis* Fairmaire 1895: 16;
*Helictopleurusgibbicollis* d’Orbigny 1915: 433 (new combination) (**syn. nov.**) (Figs 2f, 3b); Gillet and Boucomont 1927: 110; Lebis 1960: 119; Paulian 1986: 105; Montreuil 2005a: 134;

#### Materials

- **Type status:** Holotype. **Occurrence:** individualCount: 1; sex: male; lifeStage: adult; preparations: dry; disposition: in collection; otherCatalogNumbers: MALP0613; occurrenceID: 0D6427C9-F941-5E24-9379-940C5792B676; **Taxon:** scientificName: Helictopleurusclouei (Harold, 1869); namePublishedIn: Harold, E. von 1869. Coprophage Lamellicornien mit besonderer Berücksichtigung der Pariser Sammlungen, 5:46–70; class: Insecta; order: Coleoptera; family: Scarabaeidae; genus: Helictopleurus; specificEpithet: clouei; taxonRank: species; scientificNameAuthorship: Harold, 1869; nomenclaturalCode: ICZN; taxonomicStatus: valid; **Location:** continent: Africa; country: Madagascar; verbatimLocality: Madagascar; **Identification:** identifiedBy: E. von Harold; **Record Level:** institutionCode: MNHN; basisOfRecord: PreservedSpecimen
- **Type status:** Syntype. **Occurrence:** individualCount: 1; sex: male; lifeStage: adult; preparations: dry; disposition: in collection; otherCatalogNumbers: MALP0649; occurrenceID: B95EA3D8-F28F-59EF-B526-3B1B5EA5A178; **Taxon:** scientificName: Helictopleurusgibbicollis (Fairmaire, 1895); namePublishedIn: Fairmaire, L.M.H. 1895. Descriptions de quelques Coléoptères de Madagascar. Annales de la Société Entomologique de Belgique, 39:8–40; class: Insecta; order: Coleoptera; family: Scarabaeidae; genus: Helictopleurus; specificEpithet: gibbicollis; taxonRank: species; scientificNameAuthorship: Fairmaire, 1895; nomenclaturalCode: ICZN; taxonomicStatus: subjective synonym; **Location:** continent: Africa; country: Madagascar; verbatimLocality: Diego-Suarez; **Identification:** identifiedBy: Fairmaire, L.M.H.; **Event:** year: 1893; **Record Level:** institutionCode: MNHN; basisOfRecord: PreservedSpecimen

#### Diagnosis

*Helictopleurusclouei* is distinguished from other members of the *H.quadripunctatus* group by the pronotum metallic-green and shining (opaque, dark green to bluish in the other species); elytra orange to dark brown (black with a medial yellow stripe in *H.perrieri*), with poorly-defined dark spots at the base and apex (spots more distinct in *H.quadripunctatus*). Harold (1869) suggests that *H.clouei* may be related to *H.quadripunctatus*, but specimens of *H.clouei* are visibly smaller.

#### Distribution

This species occurs in northern Madagascar and is relatively common in open habitat (Fig. 3b).

#### Taxon discussion

According to Harold (1869), the original description of *H.clouei* was allegedly based on a single female specimen without exact type locality. In the present study, the examination of this unique syntype allowed us to confirm that it is actually a male. This species is today assigned to the *quadripunctatus* group, together with *H.quadripunctatus* (Olivier, 1789), *H.perrieri* (Fairmaire, 1898) and *H.gibbicollis* (Fairmaire, 1895) (Lebis 1960, Montreuil 2005a).

Amongst the numerous *Helictopleurus* authored by L. M. H. Fairmaire (1820–1906), in 1895, he described *H.gibbicollis* from a single male specimen collected by C. A. Alluaud (1861–1949) in Diego Suarez (Fairmaire 1895). This species was originally assigned to the *semivirens* group (Lebis 1960) and later moved to the *quadripunctatus* group (Montreuil 2005a). Despite Fairmaire’s explicit statement about the collecting event, it is still unclear why Lebis (1960) and Paulian (1986) indicate "Mandritsara" as type locality of the species.

Over the years and despite the intense dung beetle surveys carried out in Diego Suarez and surroundings (e.g. Hanski et al. (2007)), *H.gibbicollis* has never been recollected nor mentioned in any work. This makes the taxonomic identity of this species fairly questionable and in urgent need of validation.

The morphological examination of the syntypes of *H.clouei* and *H.gibbicollis* allowed us to establish the latter as a junior subjective synonym of *H.clouei* (*H.gibbicollis* **syn. nov.**). Probably, the different colour of the elytra of the two specimens (dark brown in the syntype of *H.gibbicollis* and bright orange in *H.clouei*) might have confused former authors, who kept considering *H.clouei* and *H.gibbicollis* as distinct species. However, the shape of the cephalic ornaments (carinas and frontal horn) and pronotum, the pronotal tegument, the colour pattern of elytra and the shape of the parameres clearly indicate that the two specimens are conspecific.

This new synonymy reduces the number of species of the *quadripunctatus* group to three.

## Discussion

### Helictopleurus: recent extinction or undiscovered synonyms?

The latest catalogue of *Helictopleurus* includes 60 species (Montreuil 2005a). Since then, new taxa have been described, bringing the total count of species and subspecies to 71 (e.g. Montreuil 2005b, Montreuil 2007, Montreuil 2011, Montreuil 2012, Rossini et al. 2021). Many *Helictopleurus* species were originally described from specimens collected in the early 20^th^ century and have not been recollected since. Notably, a significant dung beetle sampling intiative during 2002–2006 (Hanski et al. 2007) managed to collect only 29 species, representing approximately 50% of the known diversity at that time. Based on these findings, Hanski et al. (2007) suggested that the absent species might have become extinct, likely due to the widespread deforestation in Madagascar driven by human activities.

Contrasting this view, our prior and current study (Rossini et al. 2021) may indicate that species loss in *Helictopleurus* may not be as severe as previously thought. The loss of pristine forest is unquestionably one of the major threat to Madagascan biodiversity (Morelli et al. 2019, Antonelli et al. 2022). Nonetheless, the current trends in *Helictopleurus* also suggest that a number of currently-accepted species names might actually be undiscovered synonyms. Thus, the loss of 50% species diversity, attributed to extinction as proposed by Hanski et al. (2007) should be reassessed after an extensive taxonomic review of *Helictopleurus*. This casts a more optimistic light on the conservation status of *Helictopleurus* species in Madagascar and underscores the importance of thorough taxonomic studies in understanding biodiversity and extinction rates.

### Nanopublications and Taxonomy

We created the nanopublications in the nanodash platform and integrated them into this article using the ARPHA writing tool provided by Pensoft. Each of our nanopublications contains a statement like "species name X is a subjective synonym (obo:NOMEN_0000285) of species name Y, published by M. Rossini, based on this article". The identifiers for the species names are supplied by ChecklistBank. Nanopublications are written using RDF language (akin to XML) by utilising terms from established ontologies and vocabularies (Kuhn et al. 2021). As a framework for graph-based data representation on the web, RDF allows modelling of complex semantic structures, ensuring that nanopublications can be understood by computers (Dimitrova et al. 2021, Giachelle et al. 2021). Due to this flexibility, nanopublications can be used to convey a wide range of scientific information besides synonymy (see the nanodash platform for details).

Upon creation in nanodash, nanopublications are instantly accessible via a decentralised network maintained by Knowledge Pixels, which makes them available to anyone with internet access. It is important to note that nanopublications are not peer-reviewed upon creation. This approach facilitates the rapid sharing of biological data, but it also raises significant concerns within the community about the potential contamination with low-quality data. Notably, the nanodash platform distinctly labels nanopublications as either peer-reviewed or non-peer-reviewed, enabling their straightforward separation. We therefore believe that nanopublications do not seem to pose a threat to data quality or to flooding the informational landscape with undesirable content. Nevertheless, this topic requires further discussion within the scientific community.

Peer-reviewed nanopublications are directly linked to their corresponding papers, facilitating their citation. Therefore, integration of nanopublications with taxonomy shows great potential to enhance the citation and acknowledgment of taxonomic studies (Mons et al. 2011, Patterson et al. 2014). To illustrate the practical application of the nanopublications, we provided an exemplar SPARQL query. It retrieves all synonyms along with their valid names and associated metadata. SPARQL, the standard query language for Linked Open Data on the web or RDF datastores, allows constructing complex queries for various types of data.

Nanopublications streamline the traditionally labour-intensive process of updating and accessing taxonomic information. In the conventional approach, researchers must diligently surf through relevant taxonomic papers to keep data up-to-date, a task that is both time-consuming and demanding. Similarly, major data aggregators, such as GBIF or the Catalogue of Life, face challenges in extracting information from traditional publications, often relying on manual processing or use of semi-supervised tools. Besides requiring additional effort, this also leads to delays in making the new data available after they are published. Nanopublications address these challenges by making taxonomic data immediately accessible and queryable online.

There is an ongoing initiative to link nanopublications with ZooBank (International Code of Zoological Nomenclature), aiming to make taxonomic and nomenclatural information swiftly available and broadly accessible to researchers. This integration could significantly accelerate the dissemination of taxonomic knowledge across various domains of science. Generally, nanopublications hold great promise for enhancing efficiency and reducing the time and effort required in managing taxonomic data.

## Supplementary Material

XML Treatment for Helictopleurus halffteri

XML Treatment for Helictopleurus rudicollis

XML Treatment for Helictopleurus vadoni

XML Treatment for Helictopleurus clouei

92F01D0B-656C-592D-AF30-22C836C1518A 10.3897/BDJ.12.e120304.suppl1 Supplementary material 1 Biogeographic data Data type occurrences Brief description Collecting data in DwC format. File: oo_971923.csv https://binary.pensoft.net/file/971923 Rossini M., Montanaro G., Montreuil O., Tarasov S.

2B65C2E3-3B06-5886-9AFE-E9236D041C77 10.3897/BDJ.12.e120304.suppl2 Supplementary material 2 SPARQL query Data type Text file in SPARQL format File: oo_975631.sparql https://binary.pensoft.net/file/975631 Kuhn T., Tarasov S.

## Figures and Tables

**Table:** 

Nanopublication	Creator	Date

**Figure 2a.**
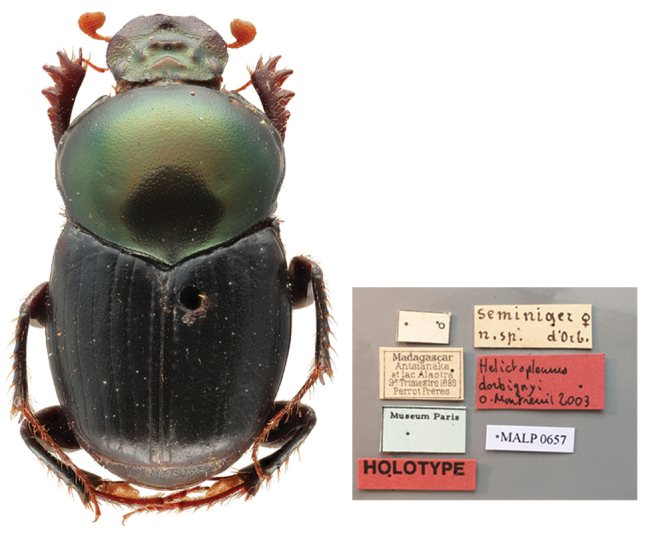
Dorsal habitus and labels of the holotype of *H.dorbignyi*;

**Figure 2b.**
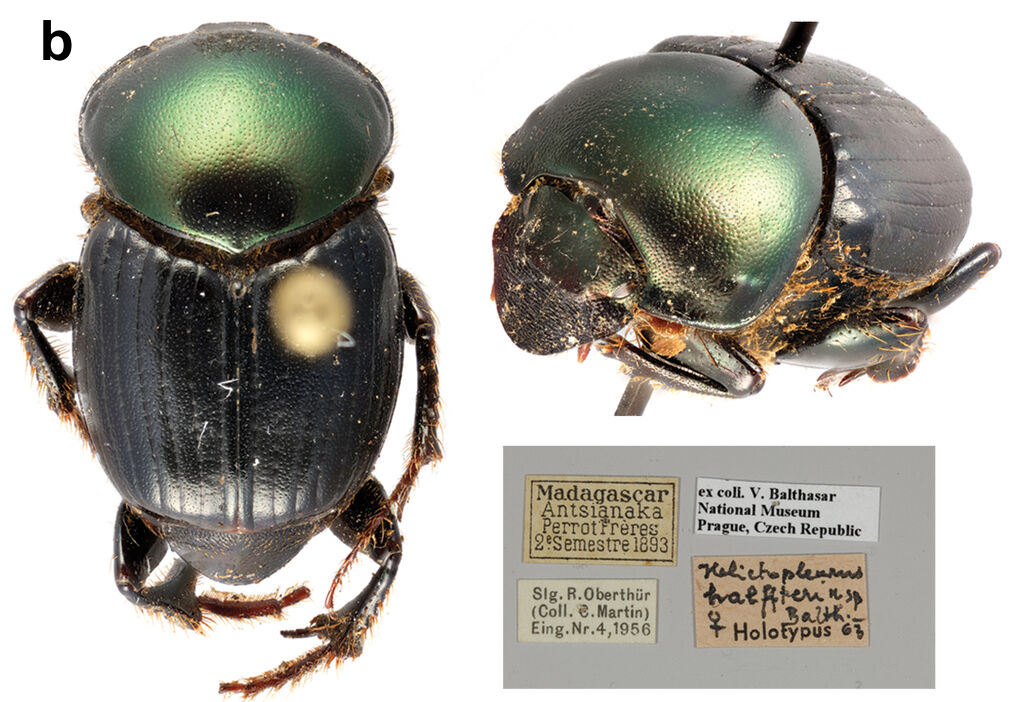
Dorsal and frontolateral habitus and labels of the holotype of *H.halffteri*;

**Figure 2c.**
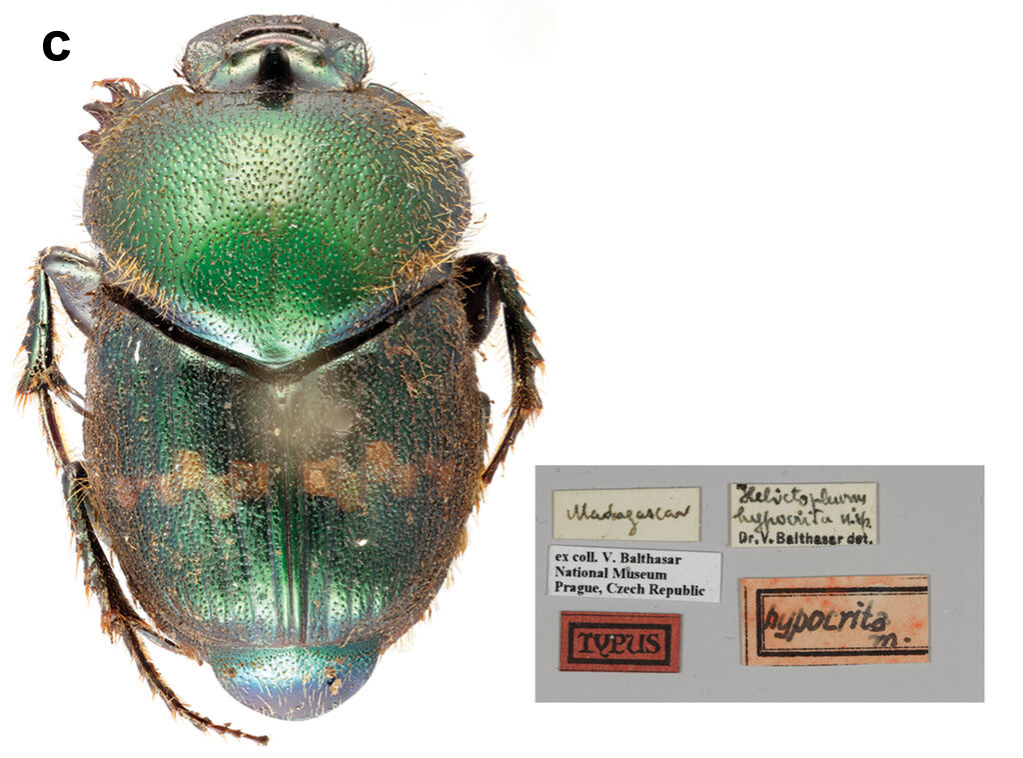
Dorsal habitus and labels of the holotype of *H.hypocrita*;

**Figure 2d.**
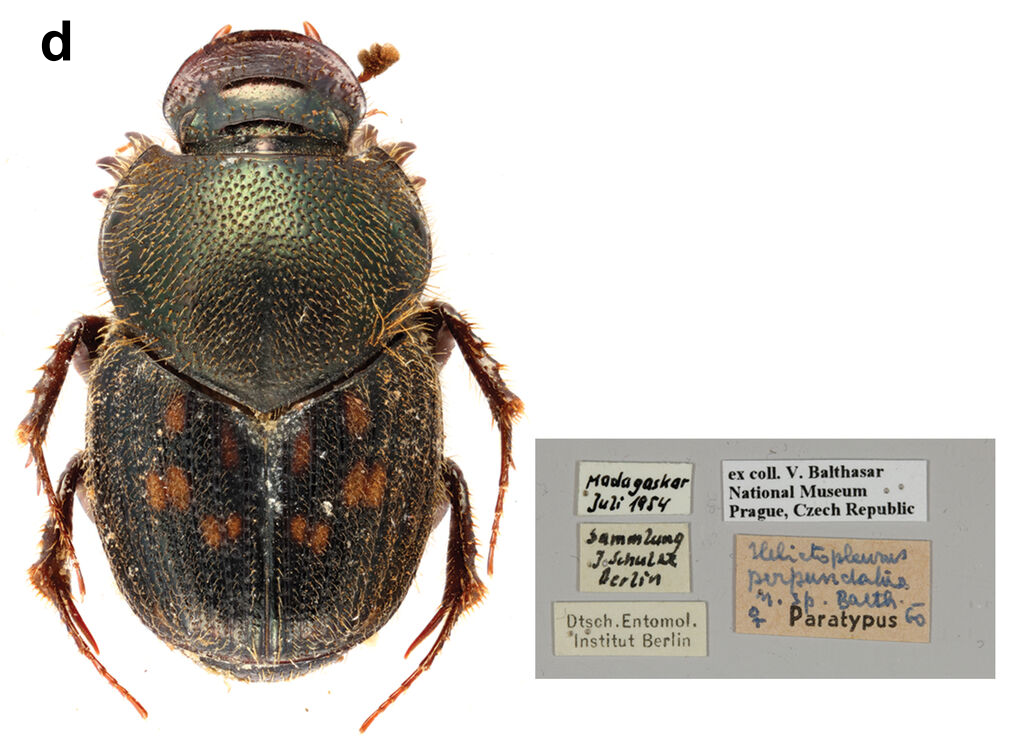
Dorsal habitus and labels of the paratype of *H.perpunctatus*;

**Figure 2e.**
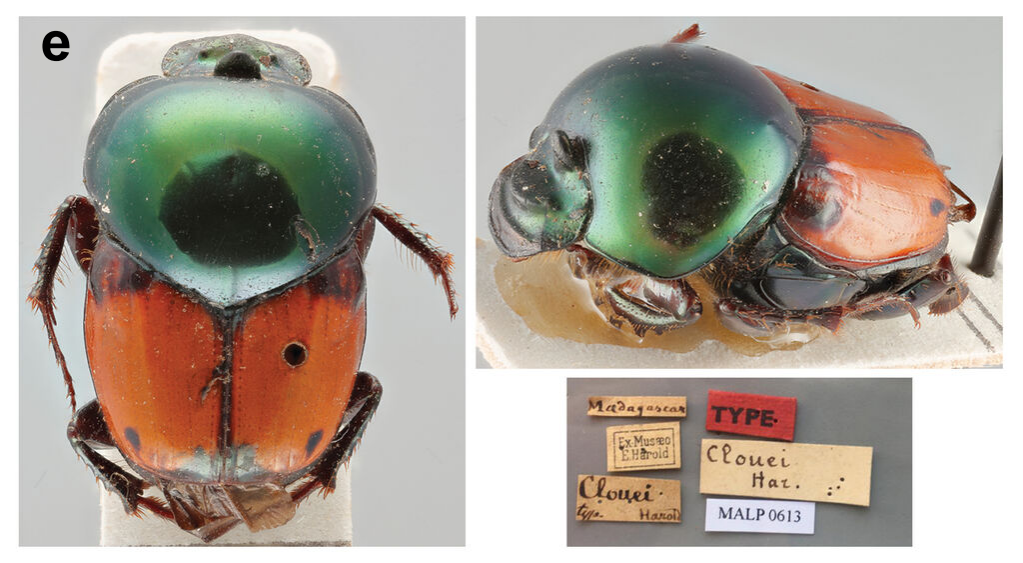
Dorsal and frontolateral habitus and labels of the syntype of *H.clouei*;

**Figure 2f.**
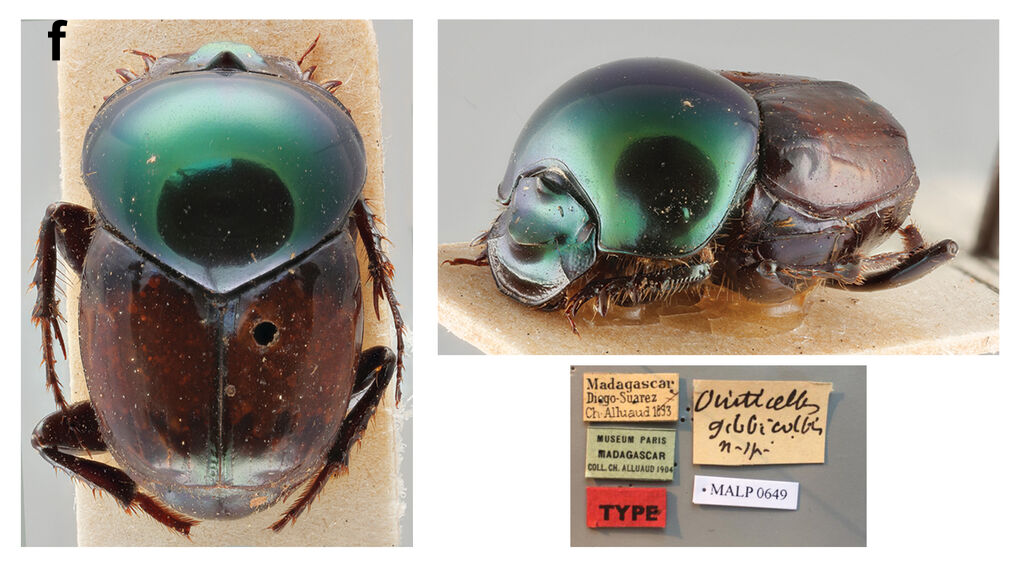
Dorsal and frontolateral habitus and labels of the syntype of *H.gibbicollis*.

**Figure 3a.**
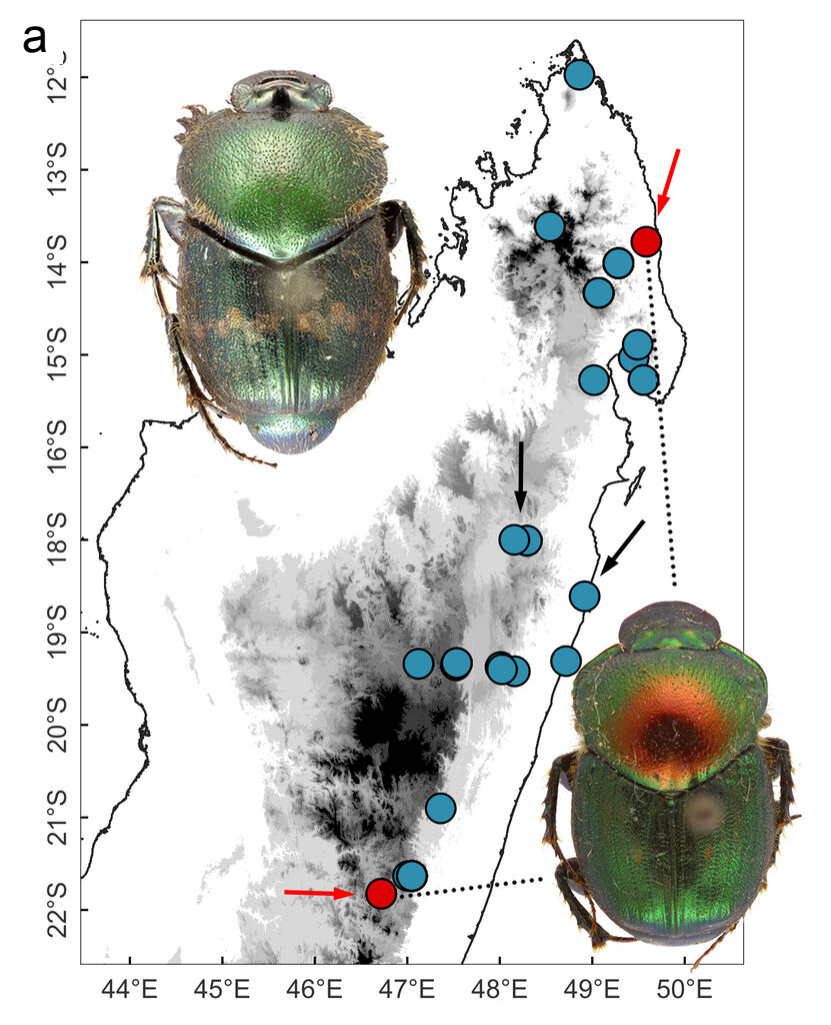
*H.rudicollis* (blue circles; black arrows indicate the type locality) and *H.splendidus* (red circles; red arrows indicate the type localities);

**Figure 3b.**
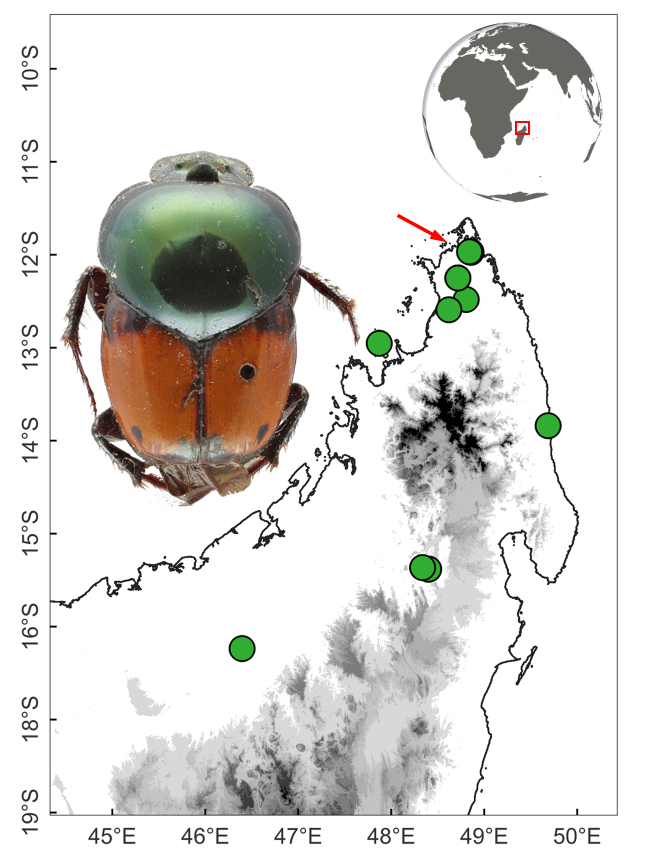
*H.clouei* (red arrow indicates the type locality of *H.gibbicollis* **syn. nov.**);

**Figure 3c.**
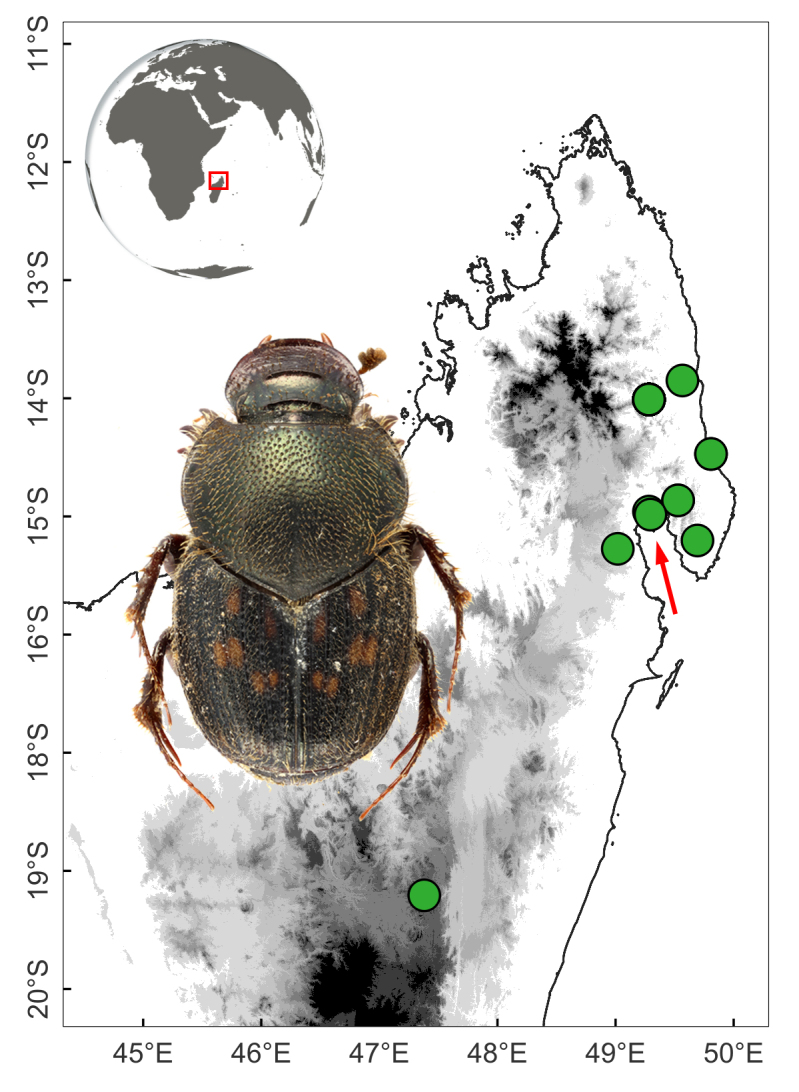
*H.vadoni* (red arrow indicates the type locality);

**Figure 3d.**
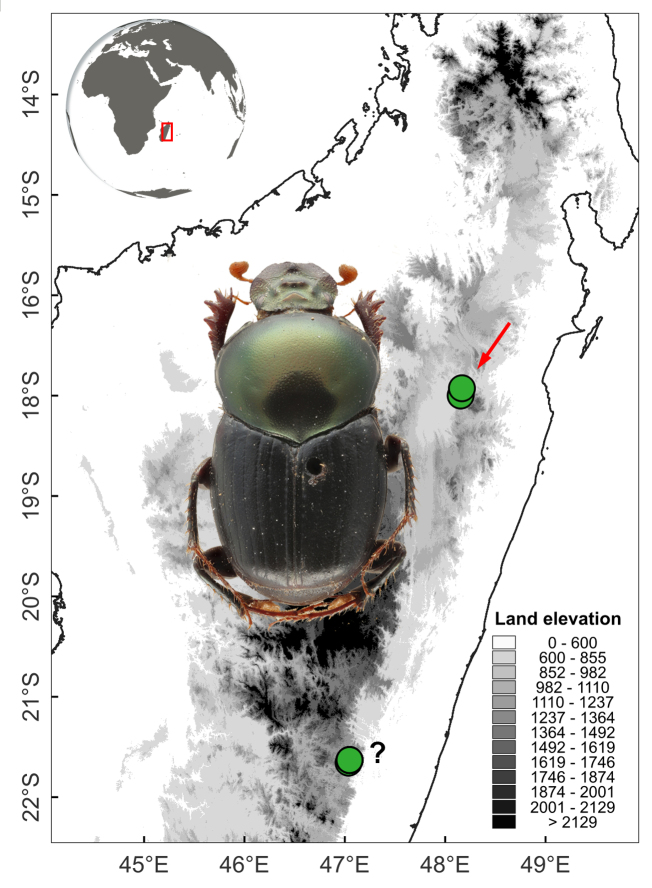
*H.halffteri* (red arrow indicates the type locality of *H.halffteri* and *H.dorbignyi*). Question mark indicates the collecting localities of some of the type specimens of *H.dorbignyi* **syn. nov.** (=*H.halffteri*). These specimens may belong to an undescribed species.
